# BDNF promoted osteoblast migration and fracture healing by up‐regulating integrin β1 via TrkB‐mediated ERK1/2 and AKT signalling

**DOI:** 10.1111/jcmm.15704

**Published:** 2020-08-16

**Authors:** Zitao Zhang, Polu Hu, Zhen Wang, Xusheng Qiu, Yixin Chen

**Affiliations:** ^1^ Department of Orthopedics The Affiliated Drum Tower Hospital of Nanjing University Medical School Nanjing China; ^2^ Nanjing Red Cross Blood Center Nanjing China

**Keywords:** brain‐derived neurotrophic factor, fracture healing, integrin β1, MC3T3‐E1 cells migration

## Abstract

Brain‐derived neurotrophic factor (BDNF) has been reported to participate in fracture healing, whereas the mechanism is still unclear. Since osteoblast migration is important for fracture healing, investigating effects of BDNF on osteoblasts migration may help to reveal its mechanism. Here, MC3T3‐E1 cells were used in vitro while closed femur fracture mice were applied in vivo. Cells migration was assessed with Transwell assay. The protein expression was analysed by immunoblotting. X‐ray and Micro‐CT were performed at different time after fracture. Our results showed that BDNF promoted MC3T3‐E1 cells migration, integrin β1 expression and ERK1/2 and AKT phosphorylation. K252a, a specific inhibitor for TrkB, suppressed BDNF‐induced migration, integrin β1 expression and activation of ERK1/2 and AKT. PD98059 (an ERK1/2 inhibitor) and LY294002 (an AKT inhibitor) both inhibited BDNF‐induced migration and integrin β1 expression while integrin β1 blocking antibody only suppressed cell migration. X‐ray and Micro‐CT analyses showed that the adenoviral carried integrin β1 shRNA group had slower fracture healing at 7 and 21 days, but not 35 days compared to the control group. Thus, we proposed that BDNF stimulated MC3T3‐E1 cells migration by up‐regulating integrin β1 via TrkB mediated ERK1/2 and AKT signalling, and this may help to enhance the fracture healing.

## 1 INTRODUCTION

Brain‐derived neurotrophic factor (BDNF), the second member of the neurotrophin family, is a major regulator of neural survival, development and function.^1^, ^2^ TrkB (tropomyosin‐related kinase B), the specific receptor of BDNF, has a unique transmembrane segment and an intracellular domain characterized by Trk activity. When interacting with BDNF, the dimerization and autophosphorylation of TrkB will happen and then the subsequent activation of signalling pathways including Ras as well as MAP kinase, PI3‐kinase and phospholipase‐Cγ (PLC‐γ) pathways is regulated.^2^, ^3^ Recent studies gradually reveal its important roles in fracture healing. Kilian O confirmed the expression of BDNF and its tropomyosin‐related kinase B receptor (TrkB) in fracture callus from human samples, while Streit revealed BDNF trended higher following adjunctive use of pulsed electromagnetic fields in fifth metatarsal fracture non‐union patients.^4^, ^5^ Titanium‐40‐niobium alloys coated with BDNF could enhance osteoblast differentiation from osteoporotic donors.^6^ Bone cement filled with BDNF could significantly promote fracture healing in animal model.^7^, ^8^ However, the underlying mechanism of BDNF in fracture healing is largely unknown.

Fracture healing is a complex physiologic process that involves several cell types. Osteoblasts are the major bone‐forming cells that play a crucial role in this process.^9^ During fracture healing, osteoblast precursor cells must migrate into fracture sites from the bone marrow compartment, where they initiate the process of fracture repair including adherence, differentiation and deposition of the bone matrix.^10^, ^11^ Many studies have shown that BDNF plays important roles in the regulation of cell migration.^12^, ^13^, ^14^, ^15^ BDNF can promote young cardiac microvascular endothelial cells to migrate via the activation of the BDNF‐TrkB‐PI3K/AKT pathway after myocardial infarction.^15^ In enhancing the generation of periodontal tissue, BDNF stimulates endothelial cell migration by a process involving TrkB/ERK/integrin signalling.^14^ In addition, BDNF also can potentiate EGF (epidermal growth factor)‐induced migration of human foetal neural stem/progenitor cells via the PI3K/AKT pathway in neurogenesis.^12^ Whether BDNF participates in fracture healing through regulating osteoblast migration remains unclear.

Integrins are transmembrane receptors composed of α subunits and β subunits assembled as heterodimers and are involved in cell‐cell and cell‐extracellular matrix interaction and integrin β1 has been demonstrated as the major subunit in osteoblasts.^10^, ^16^ Previous study has proved that bone formation is impaired resulting from altering osteoblast function in transgenic mice with a dominant‐negative integrin β1.^17^ During fracture healing, Einhorn also demonstrates callus size is obviously diminished in α1β1 integrin‐deficient mice.^18^ However, the underlying mechanism is still poorly understood. Cooperating with kinds of growth factors, integrins transduce signals from the environment into cells and often mediate cell adhesion and migration.^14^, ^19^, ^20^, ^21^ Xiong suggested TGFβ1 induced endometrial cancer cell adhesion and migration by up‐regulating integrin αvβ3 while Furmento indicated granulocyte colony‐stimulating factor (G‐CSF) up‐regulated integrin β1 and increased migration of human trophoblast Swan 71 cells.^19^, ^20^ For BDNF signalling, integrin β3 and β5 has already been proved to be up‐regulated and participate in manipulating endothelial and chondrosarcoma cells migration, respectively.^14^, ^21^ Thus, we hypothesized that there must be an existing regulatory network involving BDNF and integrin β1 in osteoblasts migration during fracture healing.

In the present study, we sought to define the role of integrin β1 in BDNF‐induced fracture healing and to identify the signalling pathway engaged by BDNF to mediate osteoblasts migration.

## 2 MATERIALS AND METHODS

### 2.1 Reagents

Anti‐mouse ERK1/2, p‐ERK1/2^thr202/tyr204^, AKT and p‐AKT^ser473^ primary antibodies, as well as PD98058 and LY294002, were purchased from Cell Signaling Technology (Danvers, Massachusetts, USA). Integrin β1 blocking antibody (P4C10) (integrin β1 BL) and Runx2 primary antibody were obtained from Novus Biologicals (Littleton, Colorado, USA). All secondary antibodies used in this study were purchased from the Beyotime Institute of Biotechnology (Songjiang, Shanghai, China). K252a was from Merck (Darmstadt, Germany). BDNF and fibronectin were from Abcam (Cambridge, MA, USA).

### 2.2 Cell culture and treatment

Murine MC3T3‐E1 cells were obtained from the American Type Culture Collection (Manassas, Virginia, USA). Cells were cultured in α‐Minimum Essential Medium (α‐MEM; Gibco, Grand Island, New York, USA) containing 10% (*v/v*) foetal bovine serum (FBS; Gibco), 1% (*v/v*) penicillin‐streptomycin solution (Gibco). The cells were maintained in a humidified incubator (Sanyo, Jencons, United Kingdom) at 37°C with 5% CO_2_. BDNF (50 ng/mL) was added to the medium only following serum starvation for 30 minutes with durations of 0, 0.5, 3, 6 and 12 hours for integrin β1 detection and 0, 15, 30, 60 and 120 minutes for kinase assay. K252a (200 nmol/L, an inhibitor of TrkB), PD98059 (25 μmol/L, an inhibitor of ERK1/2), LY294002 (50 μmol/L, an inhibitor of AKT) or integrin β1 blocking antibody (100 μg/mL) were added to the medium 30 minutes prior to the stimulation with BDNF or fibronectin (20 μg/mL). An equal quantity of dimethyl sulfoxide (DMSO) was used for incubation of the cells as a solvent control.

### 2.3 Western blot analysis

Total cellular extracts were obtained by performing cell lysis using an RIPA buffer (Beyotime Institute of Biotechnology). The protein concentration was quantified using the Bradford method. Aliquots of cell lysates were separated by 12% SDS‐polyacrylamide gel and transferred to polyvinylidene fluoride membranes (Millipore, Billerica, MA, USA). The membranes were incubated overnight with anti‐mouse ERK1/2 (1:1000), p‐ERK1/2^thr202/tyr204^ (1:1000), AKT (1:1000), p‐AKT^ser473^ (1:1000) and integrin β1 (1:500) at 4°C. This was followed by the addition of a horseradish peroxidase‐linked secondary antibody (1:1000), and electrochemiluminescence visualization of the bands (Beyotime Institute of Biotechnology). Quantification of the bands was performed using Quantity One densitometric analysis software (Bio‑Rad Laboratories, Hercules, CA, USA).

### 2.4 Transwell cell migration assay

Migration of MC3T3‐E1 cells was determined by using the Costar Transwell System (8‐μm pore size polycarbonate membrane inserts, Costar, Cambridge, MA). In brief, after different treatment, cells (1 × 10^3^ cells/200 μL FBS‐free medium) were seeded into the upper chamber and 600 μL complete medium was added into the lower chamber. After incubation at 37°C for 12 and 24 hours, non‐migrated cells in the upper chamber were removed using cotton swab carefully and cells that went through the inserts were fixed with 4% paraformaldehyde, followed by staining in crystal violet. The number of migrated cells in five randomly chosen fields was counted under a fluorescence microscope (Nikon, Tokyo, Japan).

### 2.5 Integrin β1 shRNA adenovirus production

The adenoviral vectors carried mouse integrin β1 shRNA (Ad‐integrin β1 shRNA) or the negative control (Ad‐NC) was constructed by GeneChem Co., Ltd (Shanghai, China). The shRNA sequences were as follows: Ad‐integrin β1 shRNA (5′‐AATTCGCACCAGCCCA TTTAGCTATTCAAGAGATAGCTAAATGGGCTGGTGCTTTTTTG‐3′) and Ad‐NC (5′‐GA TCCGTTCTCCGAACGTGTCACGTAATTCAAGAGATTACGTGACACGTTCGGAGAATTTTTTC‐3′).

### 2.6 Femur fracture animal model

Thirty adult male C57BL/6J mice were purchased from Shanghai SLAC Laboratory Animal Co., Ltd and were divided into two groups (15 mice/group, 5 mice/group at different postoperative time): negative control adenovirus group (Ad‐NC) and integrin β1 shRNA adenovirus group (Ad‐integrin β1 shRNA). After acclimated for one week, the 30 mice were anesthetized by intraperitoneal injection of 2.5% pentobarbital sodium at a dose of 10 mg/kg and closed femur fractures were created according to a previous report.^22^ Using aseptic surgical technique, a 3 mm medial parapatellar incision was made. The patella was dislocated to expose the femoral condyles. A stainless steel in a diameter of 0.45 mm was inserted into the femoral intramedullary canal via a retrograde approach. Then after cutting, the needle was pushed deep into the marrow cavity of the femur and the wound was closed with a 5‐0 silk suture. The animal was transferred to the collision platform of the three points bending device in a prone position with the blade above the right femur. A weight of 500 g set was released from a height of 17 cm (adjust the falling height according to the size of mice) to create a right femoral fracture. The animals were radiographed immediately to verify the creation of a mid‐diaphyseal fracture. No antibiotics were administered to mice postoperatively. After a postoperative recovery period of 24 hours, Ad‐integrin β1 shRNA (1.5 × 10^10^ pfu/kg body weight) or Ad‐NC (1.5 × 10^10^ pfu/kg body weight) with BDNF (0.1 mg/kg body weight) simultaneously suspended in 50 μL saline was slowly injected into the fracture site for two groups, respectively. After three days, the same virus with BDNF was injected again into the same site. All animal studies were performed according to the guidelines of the Animal Care and Use Committee at The Affiliated Drum Tower Hospital of Nanjing University Medical School.

### 2.7 Histological examination

After micro‐CT analysis, the fracture samples were decalcified using 15% EDTA at 25°C for 30 days. The decalcification solution was changed daily. The decalcified femora were embedded in paraffin after tissue processing and sectioned (5 mm) sagittally along the femoral shaft axis. The samples were subject to haematoxylin and eosin (H&E) staining. For the immunostaining of integrin β1 and Runx2, anti‐integrin β1 (1:50) and Runx2 (1:100) antibody and HRP‐conjugated secondary antibody were used to detect their expression in fracture callus. Peroxidase substrate DAB (Beyotime Institute of Biotechnology, Shanghai, China) was used for colour development. The ROI in the histological analysis was 2 mm below and above the fracture line. Histomorphometry measurements were quantified using ImageJ software (National Institutes of Health, USA).

### 2.8 Radiological examinations by X‐ray

At 7, 21, and 35 days after the operation, mice were killed. Radiographs of right femurs were acquired through an X‐ray device (Faxitron MX20, USA). X‐ray images were numerically scored independently by two senior orthopaedic surgeons as previously described: 0‐no callus, 1‐little to moderate callus, 2‐profuse callus, 3‐bridging callus, 4‐mature callus with intra‐fragmentary bridging and 5‐callus resorption after solid union.^23^

### 2.9 Micro‐CT analysis

After removal of soft tissues and internal nails, femurs were aligned to micro‐computed tomography scanning (Micro‐CT, Scanco Medical, Brüttisellen, Switzerland) at 7, 21 and 35 post‐operative days. Specimens were scanned at 10 μm resolution, 300 ms exposure time, 0.5 angle of increment, and 70 kV and 200 μA with the Micro‐CT scanner. Region of interest (ROI) was 2 mm proximal and 2 mm distal to the fracture line with the native cortical bone and marrow space excluded. For quantitative analysis of callus and fracture healing, Scano Evaluation software (Scano Medical) was used to obtain the data in ROI. The following morphometric parameters were evaluated: total bone volume (BV, mm^3^), total tissue volume (TV, mm^3^), callus per cent bone volume (BV/TV, %), trabecular thickness (Tb.Th, mm), trabecular separation (Tb.Sp, mm) and number of trabeculae (Tb.N, 1/mm).

### 2.10 Statistical analysis

All data were expressed as means ± standard deviation (SD) and analysed with the SPSS 20.0 software package (SPSS, USA). Statistical significance of differences between groups was calculated with an ANOVA or Student's t test. Significance was set at *P* < 0.05.

## 3 RESULTS

### 3.1 BDNF promoted MC3T3‐E1 cells migration and the expression of integrin β1

**FIGURE 1:**
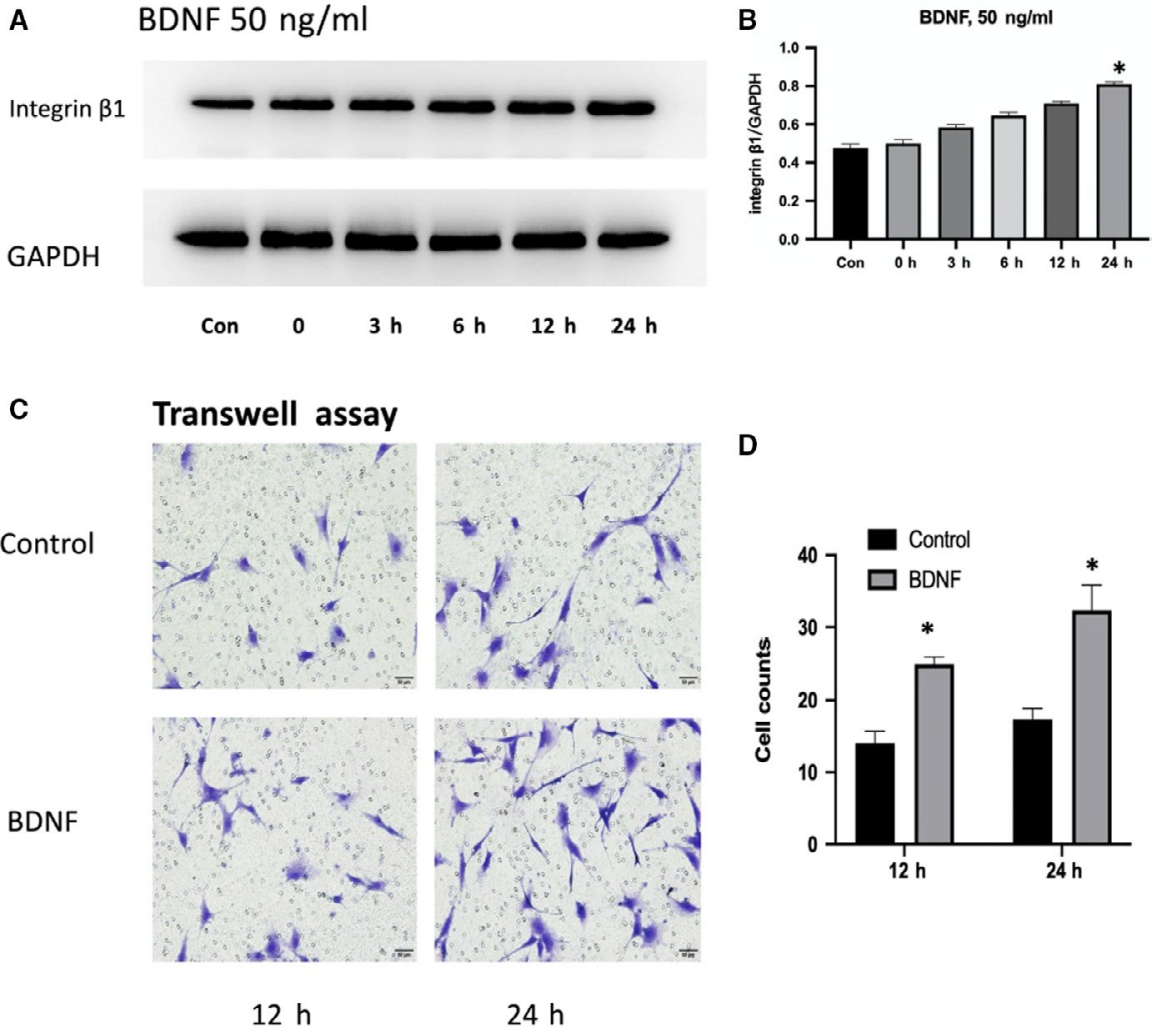
BDNF effects on the expression of integrin β1 and MC3T3‐E1 cells migration: A, MC3T3‐E1 cells were treated with BDNF (50 ng/mL) for 0, 3, 6, 12 and 24 h. The expression of integrin β1 was shown by Western blotting. B, The statistical analysis of A. C, MC3T3‐E1 cells were treated with BDNF (50 ng/mL) for 12 and 24 h. The migration of MC3T3‐E1 cells was determined by Transwell assay. D, The statistical analysis of C. Asterisks indicate statistical significance (*P < 0.05). Each experiment was repeated three times

BDNF (50 ng/mL) was used to stimulate MC3T3‐E1 cells for 0, 3, 6, 12 and 24 hours. As shown in Figure 1A, the expression of integrin β1 gradually increased and significant differences were found between 24 hours and other groups (Figure 1B). To determine whether BDNF affect migratory potential of MC3T3‐E1 cells, we performed Transwell assays for 12 and 24 hours. As shown in Figure 1B,C, BDNF played a confirmatory role in stimulating osteoblasts migration (*P* < 0.05).

### 3.2 Integrin β1 mediated BDNF‐induced MC3T3‐E1 cells migration

**FIGURE 2:**
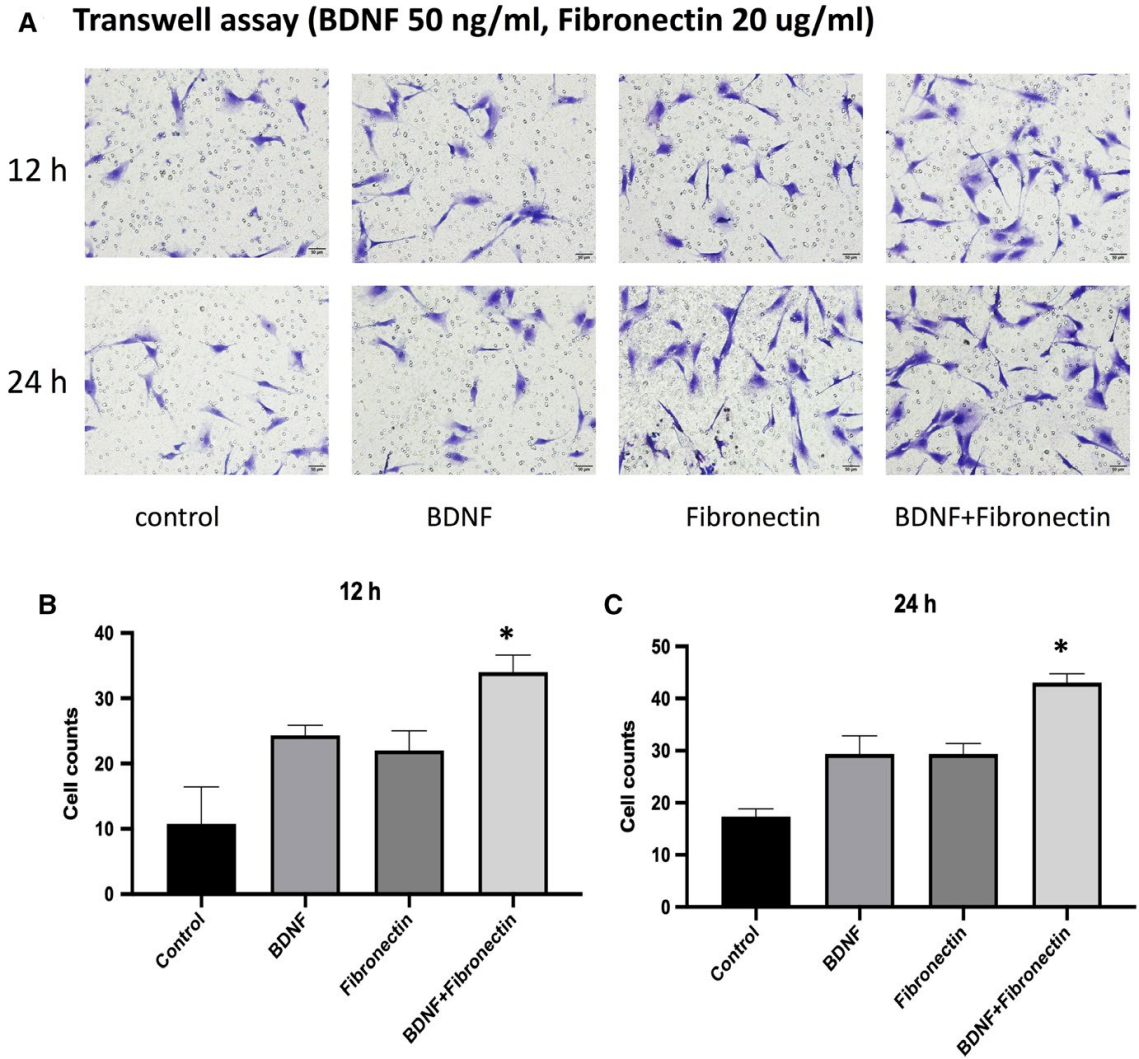
BDNF and integrin β1 effects on MC3T3‐E1 cells migration: A, Fibronectin (20 μg/mL), the most common extracellular matrix to integrin β1, was used to stimulated MC3T3‐E1 cells alone or with BDNF (50 ng/mL) for 12 and 24 h. The migration of MC3T3‐E1 cells was determined by Transwell assay. B, The statistical analysis of A for 12 h. C, The statistical analysis of A for 24 h. Asterisks indicate statistical significance with other three groups (*P < 0.05). Each experiment was repeated three times

To determine whether integrin β1 altered BDNF‐induced MC3T3‐E1 cells migration, fibronectin, the most common extracellular matrix to integrin β1, was used. MC3T3‐E1 cells were treated in BDNF combined with 20 μg/mL fibronectin or alone. When compared with the BDNF or fibronectin group, the number of migrating cells reached about 1.5‐fold by exposure to fibronectin or BDNF (Figure 2, *P* < 0.05).

### 3.3 Integrin β1 effect on BDNF‐induced MC3T3‐E1 cells migration via TrkB receptor

**FIGURE 3:**
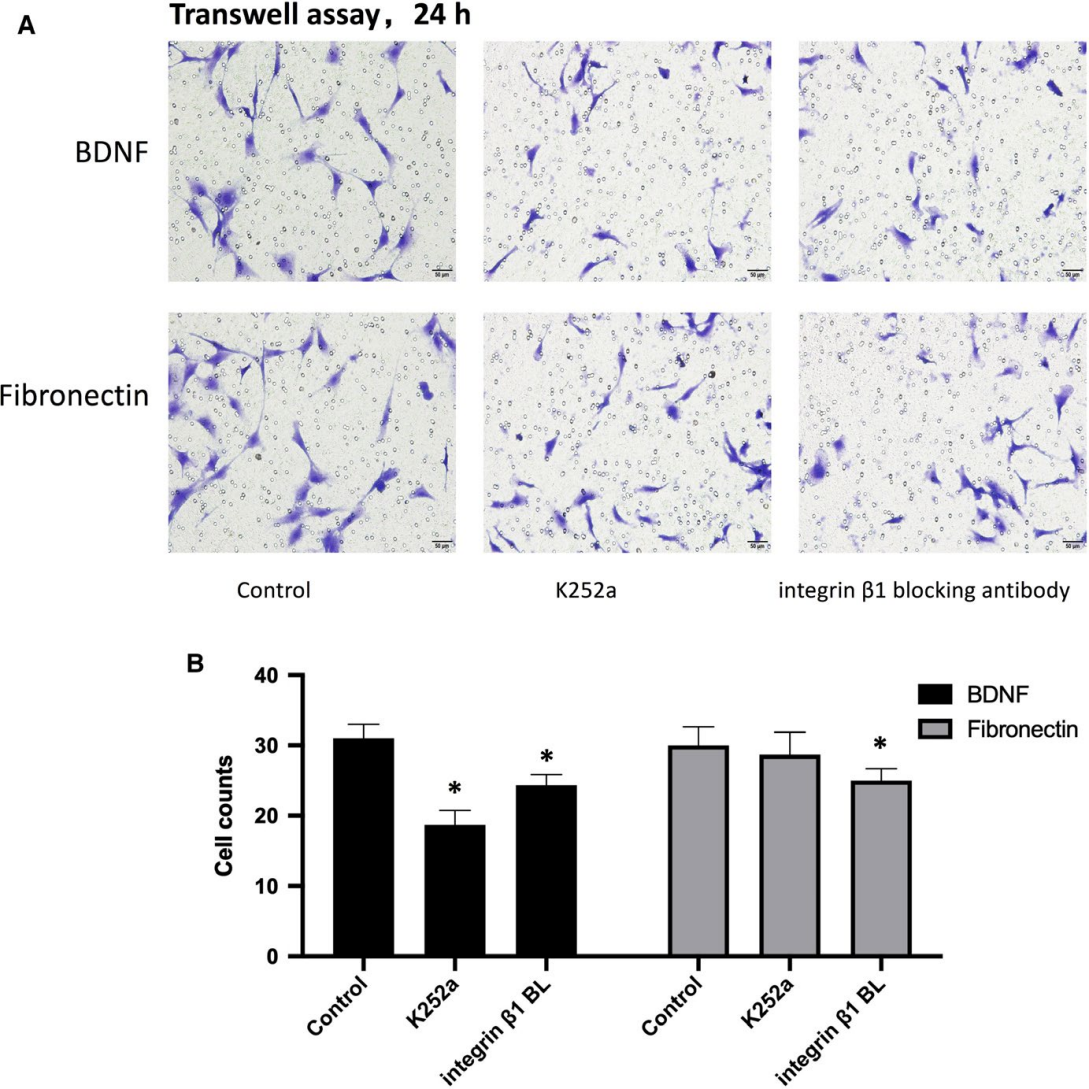
BDNF effects on MC3T3‐E1 cells migration through TrkB and integrin β1: A, BDNF (50 ng/mL) or fibronectin (20 μg/mL) was used to stimulate MC3T3‐E1 cells migration for 24 h. The TrkB receptor inhibitor K252a (200 nmol/L) or integrin β1 blocking antibody (integrin β1 BL) (100 μg/mL) was added to the medium 30 min prior to the stimulation with BDNF or fibronectin. The migration of MC3T3‐E1 cells was determined by Transwell assay. B,The statistical analysis of A for BDNF or fibronectin stimulation. Asterisks indicate statistical significance with control group (*P < 0.05). Each experiment was repeated three times

Next, we determined whether the TrkB receptor and integrin β1 were involved in BDNF‐mediated MC3T3‐E1 cells migration. Pre‐treatment of cells with the TrkB receptor inhibitor K252a and integrin β1 blocking antibody (integrin β1 BL) obviously reduced BDNF‐induced increases in cell migration (Figure 3A,B). However, when MC3T3‐E1 cells were stimulated directly by fibronectin, only integrin β1 BL could decrease the migration significantly (Figure 3A,B).

### 3.4 ERK1/2 and AKT activation were involved in BDNF‐induced cell migration and integrin β1 expression in MC3T3‐E1 cells

**FIGURE 4:**
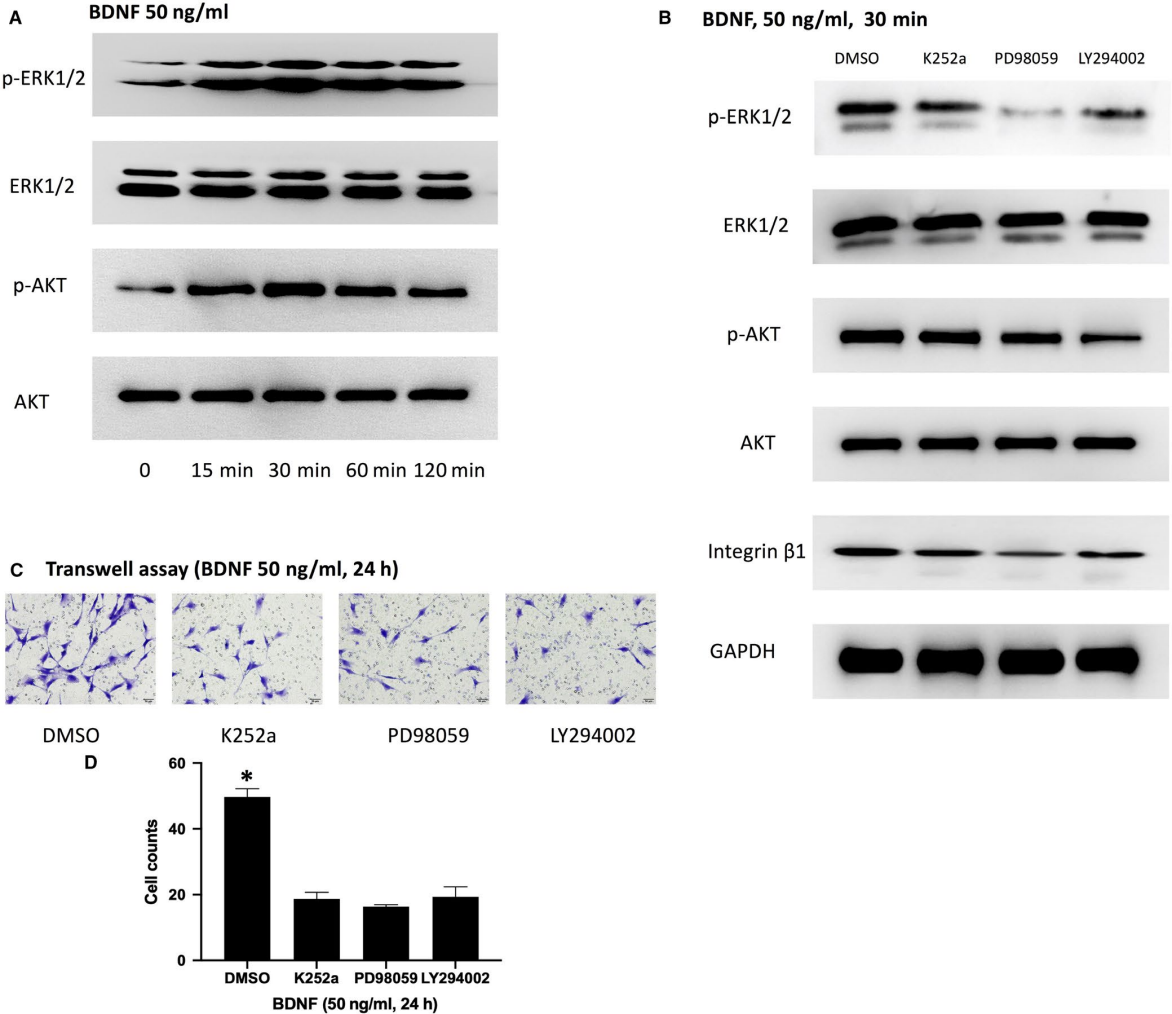
BDNF effects on MC3T3‐E1 cells migration and integrin β1 expression through TrkB‐mediated ERK1/2 and AKT activation: A, MC3T3‐E1 cells were treated with BDNF (50 ng/mL) for 0, 15, 30, 60 and 120 min. The level of p‐ERK1/2^thr202/tyr204^ and p‐AKT^S473^ was significantly increased and peaked at 30 min. B, MC3T3‐E1 cells were pretreated with K252a, PD98059 (25 μmol/L, an inhibitor of ERK1/2) and LY294002 (50 μmol/L, an inhibitor of AKT) 30 min prior to BDNF stimulation. All the three inhibitors could significantly suppress integrin β1 expression when successfully decreasing its corresponding kinase level. C, MC3T3‐E1 cells were pretreated with K252a, PD98059 and LY294002 30 min prior to BDNF stimulation. The migration of MC3T3‐E1 cells was determined by Transwell assay. D, The statistical analysis of C. Asterisks indicate statistical significance with control group (*P < 0.05). Each experiment was repeated three times

ERK1/2 and AKT is common downstream kinases in TrkB signalling.^2^, ^3^ To further confirm the involvement of ERK1/2 and AKT in BDNF effects on integrin β1 and MC3T3‐E1 cells, the level of p‐ERK1/2^thr202/tyr204^ and p‐AKT^S473^ was firstly detected. As shown in Figure 4A, the level of p‐ERK1/2^thr202/tyr204^ and p‐AKT^S473^ was significantly increased and peaked at 30 minutes. Then, the specific inhibitors including K252a for TrkB, PD98059 for ERK1/2 and LY294002 for AKT were pretreated. All the three inhibitors could significantly suppress integrin β1 expression when successfully decreasing its corresponding kinase level (Figure 4B). K252a could inhibited the activation of ERK1/2 and AKT while PD98059 and LY294002 had no effects on TrkB (Figure 4B). Furthermore, the number of migrating cells was significantly decreased when pretreated with those three inhibitors separately (Figure 4C,D). Taken together, it appears that the BDNF/TrkB axis acts through the ERK1/2 and AKT‐dependent signalling pathway to enhance integrin β1 and cell migration in MC3T3‐E1 cells.

### 3.5 Comparison of X‐ray imaging between Ad‐integrin β1 shRNA and Ad‐NC group

**FIGURE 5:**
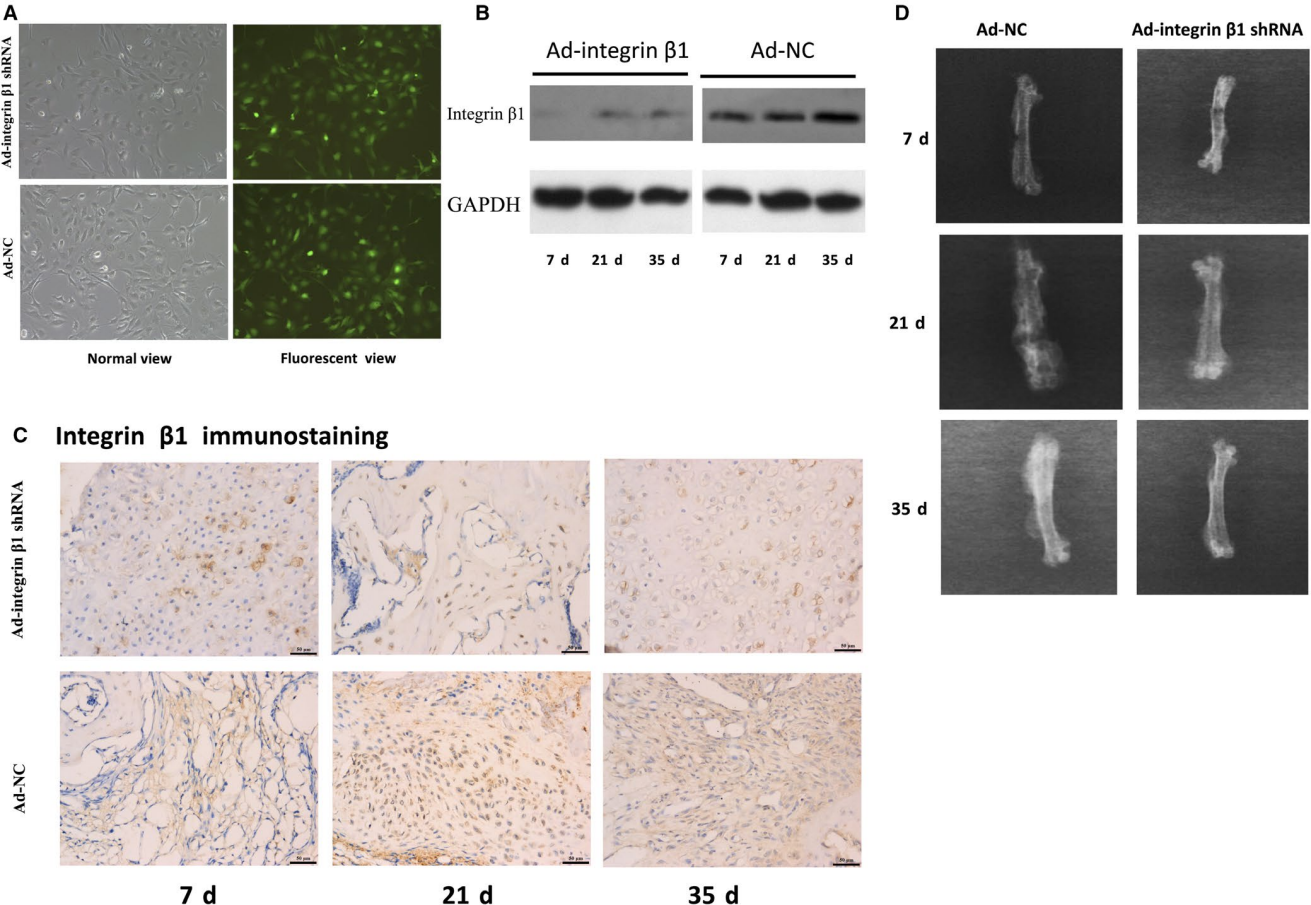
The infection efficacy of adenoviral carried integrin β1 shRNA (Ad‐integrin β1 shRNA) and adenoviral carried negative control (Ad‐NC) and representative X‐ray films of fractured femurs at 7, 21 and 35 d after fracture. A, To detect the infection efficacy of adenovirus in vitro, MCET3‐E1 cells were transfected and the green fluorescent view showed that the infected ratio was equal or greater than 95% compare to the normal view. B, The expression of integrin β1 in callus from the two groups was detected by Western blotting. The expression of integrin β1 in callus from Ad‐integrin β1 shRNA infected mice at 7, 21 and 35 d was obviously decreased compared with those in Ad‐NC groups (*P* < 0.05). C, Representative histological images of integrin β1 expression. The integrin β1 immunohistochemistry staining of callus in Ad‐integrin β1 groups at 7, 21 and 35 d were all significantly lower compared with those in Ad‐NC groups (*P* < 0.05). Scar bar = 50 μm. D, Representative X‐ray films of fractured femurs. 7 d, the two groups both showed disunion with clear fracture gaps and little periosteal callus. 21 d, the fracture lines were obscure in both the two groups, while the callus in Ad‐NC group seemed denser and bigger than that in Ad‐integrin β1 shRNA group. 35 d, the fracture line nearly disappeared and bony union was found in both the two groups

Before making radiographic and histological analysis, we first checked the infection efficacy of adenovirus in vitro and in vivo. MCET3‐E1 cells were transfected and the green fluorescent view showed that the greater than 95% infected ratio (Figure 5A). The expressions of integrin β1 in the infected femoral callus were significantly down‐regulated confirmed by Western blotting and immunohistochemistry (Figure 5B‐C).

At 7 days after operation, both Ad‐integrin β1 shRNA and Ad‐NC group showed disunion with clear fracture gaps and little periosteal callus (Figure 5D). At 21 days after operation, the fracture lines were obscure in both the two groups, while the callus in Ad‐NC group seemed denser and bigger than that in Ad‐integrin β1 shRNA group (Figure 5D). At the 35 days time‐points, the fracture line nearly disappeared and bony union was found in both the two groups (Figure 5D). However, the X‐ray score between the two groups showed no significant difference (data not shown).

### 3.6 Micro‐CT and histological analysis of fracture healing and osteoblast migration between Ad‐integrin β1 shRNA and Ad‐NC group

**FIGURE 6:**
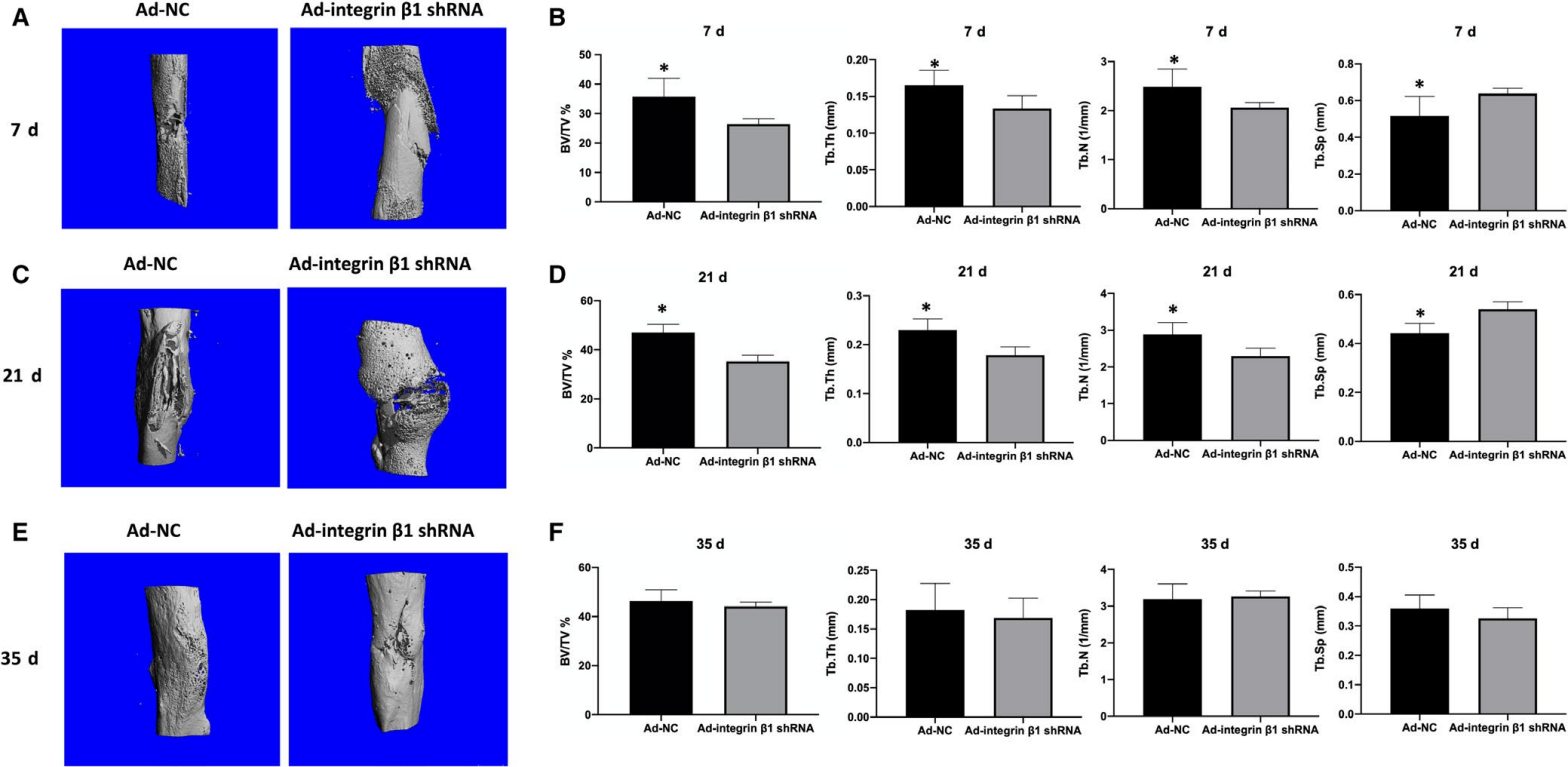
Micro‐CT analysis of fractured femoral callus from Ad‐integrin β1 shRNA and Ad‐NC group. A, Representative Micro‐CT images at 7 d after fracture. B, The statistical analysis of BV/TV, Tb.Th, Tb.Sp and Tb.N of A. C, Representative Micro‐CT images at 21 d after fracture. D, The statistical analysis of BV/TV, Tb.Th, Tb.Sp and Tb.N of C. E, Representative Micro‐CT images at 35 d after fracture. F, The statistical analysis of BV/TV, Tb.Th, Tb.Sp and Tb.N of E, Asterisks indicate statistical significance with control group (*P < 0.05). The number of mice in each group was five. BV, total bone volume; TV, total tissue volume; Tb.Th, trabecular thickness; Tb.Sp, trabecular separation; Tb.N, number of trabeculae

At 7 and 21 days after operation, micro‐CT scanning revealed a significantly lower BV/TV, Tb.Th, Tb.N and higher Tb.Sp in Ad‐integrin β1 shRNA group compared to Ad‐NC group (*P* < 0.05; Figure 6A‐D). However, no significant differences in all of the above parameters between the two groups were found in 35 days after fracture (*P* > 0.05; Figure 6E,F).

**FIGURE 7:**
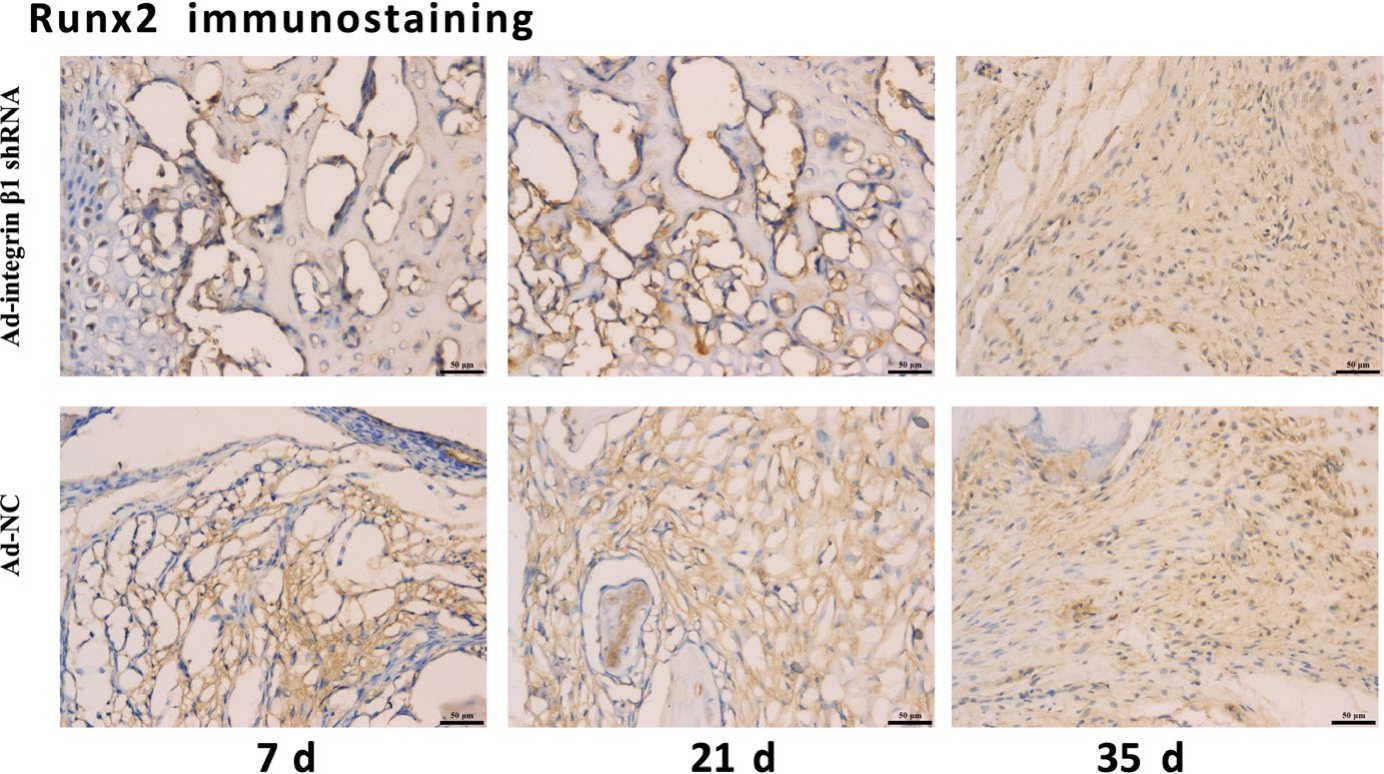
The representative histological images of Runx2 expression. Runx2, one of the specific markers of osteoblasts was used to reveal the difference of migrated osteoblasts between AD‐integrin β1 shRNA and AD‐NC group. The expression of Runx2 at 7 and 21 d after fracture in Ad‐integrin β1 group was significantly lower than that in Ad‐NC group (*P* < 0.05) while no obvious difference was found at 35 d after fracture between the two groups (*P* > 0.05). Scar bar = 50 μm

As Runx2 was one of the specific markers of osteoblasts, it was detected by callus immunohistology to find the difference in the number of osteoblasts between Ad‐integrin β1 and Ad‐NC group at 7, 21 and 35 days after fracture. As shown in Figure 7, the expression of Runx2 at 7 and 21 days after fracture in Ad‐integrin β1 group was significantly lower than that in Ad‐NC group (*P* < 0.05) while no obvious difference was found at 35 days after fracture between the two groups (*P* > 0.05).

## 4 DISCUSSION

In the present study, we demonstrated that BDNF could promote the migration and integrin β1 expression of MC3T3‐E1 cells. Integrin β1 expression induced by BDNF may be related to MC3T3‐E1 cells migration and fracture healing. Firstly, we proved that under fibronectin (the most common integrin β1 ligand) stimulation, the migratory ability of MC3T3‐E1 cells induced by BDNF was obviously enhanced (Figures 1 and 2). K252a, the specific inhibitor of TrkB, could inhibit the expression of integrin β1 induced by BDNF and decrease the number of migrating cells induced by BDNF or fibronectin, while integrin β1 blocking antibody only could suppress the migration induced by fibronectin (Figures 1 and 3). These results indicated that BDNF could promote osteoblasts migration through TrkB‐integrin β1 pathway. Then, we further investigated the role of two classical kinases ERK1/2 and AKT in BDNF/TrkB signalling. In consistent with our previous study, ERK1/2 and AKT also could be activated via BDNF‐TrkB pathway in MC3T3‐E1 cells.^24^ Inhibition ERK1/2 or AKT could significantly reduce the migration of MC3T3‐E1 cells as well as suppressing the expression of integrin β1 (Figure 4). At last, femur fracture mice also suggested that fracture healing would be perturbed especially in early and middles stage when integrin β1 shRNA was locally injected in fracture site.

Fracture repair is a complex, well‐orchestrated physiologic process that involves the activation and recruitment of bone‐forming cells, such as osteoblast precursor cells and osteoblasts.^9^ In adulthood, bone remodelling and repair require osteogenic cells to reach the sites that need to be rebuilt, as a prerequisite for skeletal health and a failure of osteoblasts to reach the sites in need of bone formation may contribute to impaired fracture repair.^25^ By using in vivo time‐lapse microscopy, Geurtzen et al demonstrated that osteoblasts started to move towards the fracture site within a single day after bone damage and that movement was due to active migration rather than passive displacement caused by cell proliferation in a bone fracture model of the zebrafish.^26^ In mouse fracture callus, Maes et al also revealed Osx‐expressing cell populations harboured, particularly in proximity to invading vasculature.^27^ In addition, some osteoblasts involved in fracture healing were systemically recruited to the fracture even from remote bone marrow sites in a rabbit ulnar osteotomy model which was adapted to trace the movement of osteogenic cells.^28^ As early as in 2000, Asaumi K firstly found neurotrophins and their receptors such as BDNF and TrkB were expressed in bone‐forming cells of mice and suggested that they were involved in the regulation of bone formation as an autocrine and paracrine factor in vivo.^29^ Afterwards, Kilian revealed that BDNF and TrkB also existed in human callus in 2014 and some clinical or experimental researches all gradually confirmed that BDNF played a positive role in accelerating bone union.^4^, ^5^, ^6^, ^7^, ^8^ However, there is little study in illustrating its mechanism. Considering the putative effects on cell migration, our study proved that BDNF/TrkB could promote osteoblasts migration for the first time. When BDNF binded to TrkB, it activated the tyrosine kinase activity of the receptor leading to trigger its intracellular signalling cascades such as ERK1/2 and AKT pathways which were two important kinases of regulating cell motility.^2^, ^13^, ^14^, ^15^, ^30^ Through TrkB/AKT signalling, BDNF regulated neuron and cardiac microvascular endothelial cells migration.^13^, ^15^ By TrkB/ERK1/2 pathway, BDNF could promote colon cancer cells and human microvascular endothelial cells migration.^14^, ^30^ To characterize downstream signalling kinases, the activation states of ERK1/2 and AKT were assessed by Western blotting at different time points. In time‐course experiments, specific phosphorylation of ERK1/2 and AKT was observed. ERK1/2 phosphorylation was inhibited by pre‐treatment with ERK1/2 inhibitor PD98059. However, treatment with AKT inhibitor LY249002 had no effect on ERK phosphorylation. Likewise, this induction of AKT phosphorylation was inhibited by pre‐treatment with LY294002, but not by PD98059. Our results revealed that activation of ERK and AKT pathways occur separately from each other without any cross talk in BDNF/TrkB‐mediated osteoblasts migration.

Integrins are α/β heterodimeric membrane receptors that regulate cellular migration, differentiation or proliferation via interacting with various extracellular matrix ligands such as fibronectin, vimentin and collagen.^16^ Integrins involvement in growth factor signalling has long been recognized.^31^ Our results showed that BDNF increased integrin β1 expression in MC3T3‐E1 cells and integrin β1 BL suppressed the BDNF‐induced migration of MC3T3‐E1 cells. Also, TrkB, ERK and AKT inhibitor suppressed the expression of integrin β1. These results suggest that integrin β1 is part of BDNF signalling cascade in controlling MC3T3‐E1 cells migration. Until now, there were only two previous researches on studying the relationship between integrins and BDNF.^14^, ^21^ In BDNF‐enhanced migration of chondrosarcoma, integrin β5 expression was up‐regulated through TrkB/AKT signalling cascade, while in BDNF‐mediated migration of microvascular endothelial cells, the up‐expression of integrin β3 was made via TrkB/ERK1/2 pathway.^14^, ^21^ Besides integrin β3 and integrin β5, our study found BDNF also could regulate integrin β1 expression for the first time and both AKT and EKR1/2 acted as two parallel downstream kinases in BDNF/TrkB signalling. As integrins often appeared as α/β heterodimeric receptors, further studies should be made to understand which α subunit was involved in BDNF‐integrin β1 controlled osteoblasts migration.

So far, a plenty of studies in vitro have confirmed that integrin β1 played a crucial role in bone formation.^10^, ^16^, ^32^ For example, optimal intensity shock wave promoted the adhesion and migration of rat osteoblasts via integrin β1 and integrin β1 mediated the BMP2 dependent transcriptional control of osteoblast differentiation and osteogenesis.^10^, ^32^ However, to some extent, the role of integrin β1 in bone formation or fracture healing was contrary in vivo. Ekholm demonstrated that callus size and cartilage synthesis was diminished in α1β1 integrin‐deficient mice during bone fracture healing.^18^ By constructing mature osteoblast specific osteocalcin‐driven transgenic mice, Zimmerman found the impaired bone formation still existed at postnatal 90 days.^17^ In the contrast, with using the same osteocalcin‐driven transgenic mice, Shekaran demonstrated that integrin β1 deletion in mature osteoblasts did not affect bone density, biomechanics and fracture healing.^33^ In our study, local injection of adenovirus, a widely used method in animal experiments, was applied. By comparing the two groups with X‐ray and micro‐CT, Ad‐integrin β1 shRNA could significantly prevent BDNF‐simulated fracture healing at 7 and 21 days, but not 35 days. Based on the present animal result, we preferred to agree more with Shekaran's study. Because at 35 days after surgery, fracture healing was in the late stage which mature osteoblasts were predominant. As integrin β1 had no effects on mature osteoblasts, it was reasonable that no differences in X‐ray and micro‐CT were found between Ad‐integrin β1 shRNA and Ad‐NC group.

Recent studies gradually proved the important role of BDNF with orthopaedic implants in fracture treatment.^6^, ^8^ Osteoblast differentiation of human mesenchymal stem cells from osteoporotic donors was significantly enhanced in presence of etched, ground titanium alloy accompanied by BDNF in vitro, while bone formation was significantly increased in osteoporotic mice that received the BDNF‐functionalized cement in the fracture gap in vivo.^6^, ^8^ To improve fracture healing rate, further studies may need to investigate the role of BDNF accompanied with different orthopaedic implants in clinically treating fracture by open reduction with internal fixation. Developing BDNF as a drug was also an alternative method in treating bone delayed union and non‐union. However, similar to other neurotrophins including nerve growth factor (NGF) and neurotrophin‐3, BDNF has poor pharmacokinetic properties, which limit its potential as a therapeutic drug. BDNF is non‐selective, susceptible to proteolytic degradation and has high molecular weight.^34^ Although some small molecule BDNF‐mimetics, such as LM22A‐4, TrkB agonist ZEB85 and 7,8‐Dihydroxyflavone, have been reported to overcome BDNF's above limitations, no studies have been made of those BDNF‐mimetics in fracture treatment.^35^, ^36^, ^37^ Recently, Johnstone et al have just demonstrated that gambogic amide, an NGF‐mimetics, appeared to have a beneficial effect on fracture healing.^38^ Therefore, the potential role of BDNF‐mimetics in fracture, including kinds, dosage and administration methods, should be further investigated.

In summary, our results suggested that BDNF contributed to MC3T3‐E1 migration and increased the expression level of integrin β1 through activation of ERK1/2 and AKT signalling pathways. Ad‐integrin β1 shRNA local administration leaded to an impairment of early and middle‐staged bone fracture healing stimulated by BDNF. These results provided further understanding for the role of BDNF with extracellular matrix in osteoblasts migration and a novel potential therapeutic candidate for fracture healing.

## CONFLICT OF INTEREST

All authors declare no conflict of interest.

## AUTHOR CONTRIBUTION

**Zitao Zhang:** Conceptualization (lead); Data curation (equal); Formal analysis (equal); Funding acquisition (equal); Investigation (lead); Methodology (equal); Project administration (lead); Resources (lead); Software (lead); Validation (equal); Visualization (equal); Writing‐original draft (lead); Writing‐review & editing (lead). **Polu Hu:** Methodology (supporting); Resources (supporting); Software (supporting). **Zhen Wang:** Investigation (supporting); Methodology (supporting). **Xusheng Qiu:** Methodology (supporting); Project administration (supporting). **Yixin Chen:** Funding acquisition (equal); Project administration (equal); Supervision (lead); Writing‐review & editing (equal).

## Data Availability

I confirm that my article contains a Data Availability Statement even if no data are available.
